# Mathematical Analysis on Current–Voltage Relations via Classical Poisson–Nernst–Planck Systems with Nonzero Permanent Charges under Relaxed Electroneutrality Boundary Conditions

**DOI:** 10.3390/membranes13020131

**Published:** 2023-01-19

**Authors:** Yiwei Wang, Lijun Zhang, Mingji Zhang

**Affiliations:** 1 College of Mathematics and Systems Science, Shandong University of Science and Technology, Qingdao 266590, China; 2 Department of Mathematics, New Mexico Institute of Mining and Technology, Socorro, NM 87801, USA

**Keywords:** PNP, permanent charges, I–V relations, critical potentials, boundary layers, 34A26, 34B16, 34D15, 37D10, 92C35

## Abstract

We focus on a quasi-one-dimensional Poisson–Nernst–Planck model with small permanent charges for ionic flows of two oppositely charged ion species through an ion channel. Of particular interest is to examine the dynamics of ionic flows in terms of I–V (current–voltage) relations with boundary layers due to the relaxation of neutral conditions on boundary concentrations. This is achieved by employing the regular perturbation analysis on the solutions established through geometric singular perturbation analysis. Rich dynamics are observed, particularly, the nonlinear interplays among different physical parameters are characterized. Critical potentials are identified, which play critical roles in the study of ionic flows and can be estimated experimentally. Numerical simulations are performed to further illustrate and provide more intuitive understandings of our analytical results.

## 1. Introduction

Ion channels are pore-forming membrane proteins allowing charged particles to pass through the channel pore. Ion channels are embedded in cell membranes, which provide a major medium for cells to communicate with each other and with outside environment ([1,2,3]). In this way, ion channels control a wide range of biological functions, in particular, many varied functions are necessary for life (see [2] for more discussion). Clinically, malfunctioning channels cause cystic fibrosis, cholera, neuronal disorders, and many other diseases ([4]). Therefore, it is significant to explore the mechanism of ion channels.

The study of ion channels generally consists of two related major topics: structures of ion channels and ionic flow properties. The physical structure of ion channels is defined by the channel shape and the spacial distribution of permanent charges and the polarity of these charges. For open channels with given structures, the main interest is in the study of its electrodiffusion property. The most challenging part in examining the properties of ionic flows through membrane channels is the characterization of the nonlinear interplays among specific physical parameters involved in the system, particularly, the boundary concentrations and membrane potential, permanent charge distribution within the channel, channel geometry and diffusion coefficients. On the other hand, all present experimental measurements about ionic flow are of input-output type ([1]); that is, the internal dynamics within the channel cannot be measured with the current technology. Therefore, it is extremely difficult to extract coherent properties or to formulate specific characteristic quantities from the experimental measurements.

Mathematical analysis plays important and unique roles for generalizing and understanding the principles that allow control of electrodiffusion, explaining the mechanics of observed biological phenomena and for discovering new ones, under the assumption that a more or less explicit solution of the associated mathematical model can be obtained. Recently, there have been some successes in mathematical analysis of Poisson–Nernst–Planck (PNP) models for ionic flows through membrane channels ([5,6,7,8,9,10,11,12,13,14,15,16,17,18,19,20,21,22,23,24,25,26] etc.). Particularly, for those that were studied under the dynamical system framework of geometric singular perturbation analysis, interesting phenomena of ionic flows were observed for relatively simple setups. However, for all the works mentioned here, the so-called electroneutrality boundary conditions are reinforced, and the boundary layers, which play critical roles in the study of ion channel problems, disappear.

To further understand the correlations/interactions among ions, we consider a PNP model with nonzero but small permanent charges under relaxed electroneutrality boundary concentration conditions. Of particular interest is the effect on the I–V relations from boundary layers, which can be mathematically extracted from solutions of the PNP system. Our study will take great advantage of the work conducted in [11]. To be specific, in [11], the authors treat the nonzero permanent charge as a small parameter, and employ the regular perturbation analysis to the solutions, from which they obtain the zeroth-order and first-order individual fluxes under electroneutrality boundary conditions. This will be our starting point. More precisely, the individual fluxes obtained in [11] allow us to define the current–voltage (I–V) relations directly (see (15) in Section 2.2). To examine the boundary layer effects on the I–V relations, we relax the electroneutrality boundary conditions by introducing two positive parameters, σ and ρ close to but not simultaneously equal to 1 (see our discussion in Section 1.3). We next employ the regular perturbation analysis to these two parameters, in other words, we expand our I–V relations at (σ,ρ)=(1,1) up to the first order and neglect higher order terms. Our main interest is then in the first-order terms, the leading terms that contain boundary layer effects.

### 1.1. One-Dimensional Poisson–Nernst–Planck Models

PNP system is a basic macroscopic model for electrodiffusion of charges through ion channels ([27,28,29,30,31,32,33,34,35,36], etc.) Under various reasonable conditions, the PNP system can be derived as a reduced model from molecular dynamics, Boltzmann equations, and variational principles ([37,38,39,40]).

Based on the fact that the channel is narrow and one can effectively view it as a one-dimensional channel [0,l], where *l* (with unit nm) is the length of the channel together with the baths that the channel links. A quasi-one-dimensional *steady-state* PNP model for a mixtures of *n* ion species though a single channel reads (first proposed in [41])

(1) $$\begin{array}{c} \begin{array}{cc} \; & \frac{1}{h(x)}\frac{d}{dx}\left(\varepsilon_{r}\left(x\right)\varepsilon_{0}h\left(x\right)\frac{d\Phi }{dx}\right)=-e\left(\sum_{s=1}^{n}z_{s}c_{s}+Q\left(x\right)\right),\\ \; & \frac{dJ_{k}}{dx}=0,\;-J_{k}=\frac{1}{k_{B}T}D_{k}\left(x\right)h\left(x\right)c_{k}\frac{d\mu_{k}}{dx},\;k=1,2,\ldots ,n, \end{array} \end{array}$$

where x∈[0,1] is the coordinate along the axis of the channel that is normalized to [0,1], h(x) is the area of cross-section of the channel over the location *x*.

For system (1), we have the following boundary conditions (see [8] for a reasoning), for k=1,2,…,n,

(2) $$\Phi \left(0\right)=V,\;\;c_{k}\left(0\right)=L_{k}>0;\;\Phi \left(1\right)=0,\;\;c_{k}\left(1\right)=R_{k}>0,\;k=1,2,\ldots ,n,$$

where

- $e\approx 1.60\times 10^{-19}$ (C = coulomb) is the elementary charge;
- $k_{B}\approx 1.38\times 10^{-23}$ (JK${}^{-1}$) is the Boltzmann constant;
- *T* is the absolute temperature (unit K (kelvin)), it is T=273.16 (K);
- Φ(x) is the electric potential with the unit V = Volt = JC${}^{-1}$;
- Q(x) is the permanent charge density of the channel (with unit 1/m${}^{3}$);
- $\varepsilon_{0}\left(x\right)$ is the local dielectric coefficient (with unit Fm${}^{-1}$);
- $\varepsilon_{r}\left(x\right)$ is the relative dielectric coefficient (with unit 1);
- h(x) represents the area of the cross-section over the point *x* (with unit m${}^{2}$);
- *n* is the number of distinct types of ion species (with unit 1);
- for the *j*th ion species;
  - $c_{j}$ is the *number* density (with unit 1/m${}^{3}$);
  - $z_{j}$ is the valence (the number of charges per particle with unit 1);
  - $\mu_{j}$ is the electrochemical potential (with unit J = CV);
  - $J_{j}$ is the *number* flux density (with unit 1/s) – the *number* of particles across each cross-section per unit time;
  - $D_{j}\left(x\right)$ is the diffusion coefficient (with unit m${}^{2}/$s).

### 1.2. Permanent Charges

It is known that the spatial distribution of side chains in a specific channel defines the permanent charge of the channel. Although some information could be obtained without considering the permanent charge and by focusing on the effects of other system parameters, such as boundary conditions, ion valences, ion sizes, etc., we believe that different channel types differ mainly in the distribution of permanent charge ([2]). To better understand the dependence of ionic flows on permanent charges, we demonstrate that the role of permanent charges in membrane channels is similar to the role of doping profiles in semiconductor devices. Semiconductor devices are similar to membrane channels in the way that they both use atomic-scale structures to control macroscopic flows from one reservoir to another. Ions move a lot like quasi-particles move in semiconductors. Roughly, holes and electrons are the cations and anions of semiconductors. Semiconductor technology depends on the control of migration and diffusion of quasi-particles of charge in transistors and integrated circuits. Doping is the process of adding impurities into intrinsic semiconductors to modulate its electrical, optical, and structural properties ([42,43]). Roughly speaking, one may understand in the following sense, doping provides the charges that acid and basic side chains provide in a protein channel. There is no doubt that, for both ion channels and semiconductors, permanent charges add an additional component—probably the most important one—to their rich behavior. In general, the permanent charge Q(x) is modeled by a piecewise constant function, that is, we assume, for a partition $x_{0}=0<x_{1}<\cdots <x_{m-1}<x_{m}=l$ of [0,l] into *m* subintervals, $Q\left(x\right)=Q_{j}$ for $x\in (x_{j-1},x_{j})$ where $Q_{j}$’s are constants with $Q_{1}=Q_{m}=0$ (the intervals $\left[x_{0},x_{1}\right]$ and $\left[x_{m-1},x_{m}\right]$ are viewed as the reservoirs where there is no permanent charge).

In [8], under the framework of geometric singular perturbation theory, the existence and uniqueness (local) was established for the boundary value problem (1) and (2) with one cation and one anion and the permanent charge function modeled by

(3) $$Q\left(x\right)=0\;\mathrm{if}\;0<x<a;\;Q\left(x\right)=Q_{0}\;\mathrm{if}\;a<x<b;\;Q\left(x\right)=0\;\mathrm{if}\;b<x<1,$$

where $Q_{0}$ is some nonzero constant. Due to the challenge in obtaining explicit expressions of the I–V relation with nonzero permanent charges, in [11], the author studied the case with $Q_{0}$ in (3) being small and employed regular perturbation analysis (viewing $Q_{0}$ as a small perturbation to the solutions of the system (1) and (2)) to further study the effects on ionic flows from the permanent charges. The analysis in [11] (Proposition 4.11 and its following discussion) indicates that *to optimize the effect of the permanent charge, a short and narrow neck within which the permanent charge is confined, is expected*. This indicates the critical role that the permanent charge plays in the study of ionic flow properties of interest.

### 1.3. Relaxed Electroneutrality Boundary Conditions

To describe the actual behavior of channels or useful transistors, macroscopic reservoirs linked by ion channels must be included ([30,31,44,45]). Macroscopic boundary conditions that describe such reservoirs introduce boundary layers of concentration and charge. If those boundary layers reach into the part of the device that performs atomic control, they prominently influence its behavior. Particularly, boundary layers of charge are probably to produce artifacts over long distances because the electric field spreads a long way.

The boundary layer should be handled more carefully in the study of such problems, particularly for ion channel problems. In [8,13,26], the boundary layer is characterized partially, mainly in establishing the existence and local uniqueness result of the PNP system. However, the effects from the boundary layers on ionic flows, which in general carry more rich information, are not analyzed. This is because, very often, when examine the qualitative properties of ionic flows through ion channels, electroneutrality boundary conditions are naturally enforced at both ends of the channel (see, e.g., [5,6,11,12,20,21,46,47,48,49,50,51]). They are defined as

(4) $$\begin{array}{c} \sum_{s=1}^{n}z_{s}L_{s}=\sum_{s=1}^{n}z_{s}R_{s}=0. \end{array}$$

However, under the condition (4), the two boundary layers disappear (see [52] for more detailed discussion).

To better understand the mechanism of ionic flows through membrane channels, the boundary layer effects should be carefully considered during the study. Meanwhile, due to the sensitivity of electric potentials on boundary layers, a first but natural step is to study the state that is not neutral but close to. More precisely, one may assume (taking n=2 in (4), for example)

(5) $$\begin{array}{c} \frac{-z_{2}L_{2}}{z_{1}L_{1}}=\sigma \;\mathrm{and}\;\frac{-z_{2}R_{2}}{z_{1}R_{1}}=\rho , \end{array}$$

where σ and ρ are some positive constants close to but not equal to 1 simultaneously (σ=1=ρ in (5) implies neutral state). Following this idea, some recent works (see [22,52,53] for examples) have shown that more rich qualitative properties of ionic flows were observed while boundary layers are involved. Particularly, the authors in [52] analyzed the boundary layer effects on individual fluxes via PNP system with nonzero but small permanent charges. All the works indicate the importance of the role played by the boundary layer in the study of ionic flow properties of interest.

### 1.4. Problem Set-Up

In this work, we take the same setting as that in [11] but *without* assuming electroneutrality boundary conditions (4) for n=2, which includes:

(A1). We consider two charged particles (n=2) with $z_{1}>0$ and $z_{2}<0$;
(A2). The PNP model only includes the ideal component $\mu_{i}^{id}\left(X\right)$ of the electrochemical potential defined by
  (6) $$\mu_{k}^{id}\left(x\right)=z_{k}e\Phi \left(x\right)+k_{B}T\ln\frac{c_{k}\left(x\right)}{c_{0}},$$
  where $c_{0}$ is some characteristic number density.
(A3). $\varepsilon_{r}\left(X\right)=\varepsilon_{r}$ and $D_{i}\left(X\right)=D_{i}$.

We will assume (A1)–(A3) from now on. We first make the following dimensionless rescaling ([11]). Let

$$\phi =\frac{e}{k_{B}T}\Phi ,\;V=\frac{e}{k_{B}T}V,\;\varepsilon^{2}=\frac{\varepsilon_{r}\varepsilon_{0}k_{B}T}{e^{2}},\;J_{k}=\frac{J_{k}}{c_{0}D_{k}},$$

Correspondingly, the boundary value problem (1) and (2) becomes

(7) $$\begin{array}{c} \begin{array}{cc} \; & \frac{\varepsilon^{2}}{h(x)}\frac{d}{dx}\left(h\left(x\right)\frac{d}{dx}\phi \right)=-z_{1}c_{1}-z_{2}c_{2}-Q\left(x\right),\\ \; & h\left(x\right)\frac{dc_{k}}{dx}+z_{k}h\left(x\right)c_{k}\frac{d\phi }{dx}=-J_{k},\;\frac{dJ_{k}}{dx}=0,\;k=1,2 \end{array} \end{array}$$

with the boundary conditions

(8) $$\phi \left(0\right)=V,\;c_{k}\left(0\right)=L_{k};\;\phi \left(1\right)=0,\;c_{k}\left(1\right)=R_{k},\;k=1,2.$$

## 2. Methods

We take the advantage of the work done in [11], and further employ regular perturbation analysis on the parameters σ and ρ introduced in (5).

### 2.1. Previous Results

To get started, we first recall some results from [11], which are fundamental for our following discussion. Treating the nonzero permanent charge $|Q_{0}|$ small compared to the boundary concentrations $L_{k}$’s and $R_{k}$’s, the authors in [11] expanded the individual flux $J_{k}\left(V;Q_{0}\right)$ along $Q_{0}=0$:

(9) $$\begin{array}{c} J_{k}\left(V;Q_{0}\right)=J_{k0}\left(V\right)+J_{k1}\left(V\right)Q_{0}+o\left(Q_{0}\right), \end{array}$$

where $J_{k}=D_{k}J_{k}$. It follows that $J_{k0}=D_{k}J_{k0}$ and $J_{k1}=D_{k}J_{k1}$, where

(10) $$\begin{array}{c} \begin{array}{cc} J_{10}= & \frac{\left(c_{1}^{L}-c_{1}^{R}\right)\left(z_{1}V+\ln L_{1}-\ln R_{1}\right)}{H\left(1\right)\left(\ln c_{1}^{L}-\ln c_{1}^{R}\right)},\;J_{20}=\frac{\left(c_{2}^{L}-c_{2}^{R}\right)\left(z_{2}V+\ln L_{2}-\ln R_{2}\right)}{H\left(1\right)\left(\ln c_{2}^{L}-\ln c_{2}^{R}\right)},\\ J_{11}= & \frac{A(z_{2}\left(1-B\right)\lambda +1)}{\left(z_{1}-z_{2}\right)H\left(1\right)}\left(z_{1}\lambda +1\right),\;J_{21}=\frac{A(z_{1}\left(1-B\right)\lambda +1)}{\left(z_{2}-z_{1}\right)H\left(1\right)}\left(z_{2}\lambda +1\right), \end{array} \end{array}$$

with

(11) $$\begin{array}{c} \begin{array}{cc} \lambda = & \frac{\phi^{L}-\phi^{R}}{\ln c_{1}^{L}-\ln c_{1}^{R}},\;A=\frac{\left(c_{1}^{L}-c_{1}^{R}\right)\left(c_{10}^{b}-c_{10}^{a}\right)}{c_{10}^{a}c_{10}^{b}\left(\ln c_{1}^{L}-\ln c_{1}^{R}\right)},\\ B= & \frac{\ln c_{10}^{b}-\ln c_{10}^{a}}{A}=\frac{\left(\ln c_{1}^{L}-\ln c_{1}^{R}\right)\left(\ln c_{10}^{b}-\ln c_{10}^{a}\right)}{\left(c_{1}^{L}-c_{1}^{R}\right)\left(c_{10}^{b}-c_{10}^{a}\right)}c_{10}^{a}c_{10}^{b}. \end{array} \end{array}$$

Here,

(12) $$\begin{array}{c} \begin{array}{cc} \; & \phi^{L}=V-\frac{1}{z_{1}-z_{2}}\ln\frac{-z_{2}L_{2}}{z_{1}L_{1}},\;z_{1}c_{1}^{L}=-z_{2}c_{2}^{L}={\left(z_{1}L_{1}\right)}^{\frac{-z_{2}}{z_{1}-z_{2}}}{\left(-z_{2}L_{2}\right)}^{\frac{z_{1}}{z_{1}-z_{2}}},\\ \; & \phi^{R}=-\frac{1}{z_{1}-z_{2}}\ln\frac{-z_{2}R_{2}}{z_{1}L_{1}},\;z_{1}c_{1}^{R}=-z_{2}c_{2}^{R}={\left(z_{1}R_{1}\right)}^{\frac{-z_{2}}{z_{1}-z_{2}}}{\left(-z_{2}R_{2}\right)}^{\frac{z_{1}}{z_{1}-z_{2}}},\\ \; & c_{10}^{a}=c_{1}^{L}+\alpha \left(c_{1}^{R}-c_{1}^{L}\right),\;c_{10}^{b}=c_{1}^{L}+\beta \left(c_{1}^{R}-c_{1}^{L}\right), \end{array} \end{array}$$

where, with $H\left(x\right)=\int_{0}^{x}\frac{1}{h(s)}ds$,

(13) $$\begin{array}{c} \alpha =\frac{H(a)}{H(1)}\;\mathrm{and}\;\beta =\frac{H(b)}{H(1)}. \end{array}$$

We define the following function, which will be used often in our analysis. For t>0, set

(14) $$\begin{array}{c} \gamma \left(t\right)=\frac{t\ln t-t+1}{(t-1)\ln t}\;\mathrm{for}\;t\neq 1\;\mathrm{and}\;\gamma \left(1\right)=\frac{1}{2}. \end{array}$$

One establishes easily that

**Lemma** **1.** *For t>0, one has*

$$0<\gamma \left(t\right)<1,\;\gamma^{\prime }\left(t\right)>0,\;\lim_{t\to 0}\gamma \left(t\right)=0\;and\;\lim_{t\to \infty }\gamma \left(t\right)=1.$$

### 2.2. Main Interest and Regular Perturbation Analysis

The most basic function of membrane channels is to regulate the permeability of membranes for a given species of ions and to select the types of ions and to facilitate and modulate the diffusion of ions across cell membranes. Currently, the permeation and selectivity properties of ion channels are usually characterized by the I–V relations measured experimentally [27,54]. Our main interest is to analyze the qualitative properties of the I–V relations and characterize the nonlinear interplays between physical parameters. More precisely, we consider

(15) $$\begin{array}{c} I\left(V;\varepsilon ,Q_{0}\right)=I_{0}\left(V\right)+I_{1}\left(V;\lambda \right)Q_{0}+o\left(Q_{0}\right), \end{array}$$

where

$$\begin{array}{c} \begin{array}{cc} I_{0}= & z_{1}D_{1}J_{10}+z_{2}D_{2}J_{20}\\ = & \frac{z_{1}D_{1}\left(c_{1}^{L}-c_{1}^{R}\right)\left(z_{1}V+\ln L_{1}-\ln R_{1}\right)}{H\left(1\right)\left(\ln c_{1}^{L}-\ln c_{1}^{R}\right)}+\frac{z_{2}D_{2}\left(c_{2}^{L}-c_{2}^{R}\right)\left(z_{2}V+\ln L_{2}-\ln R_{2}\right)}{H\left(1\right)\left(\ln c_{2}^{L}-\ln c_{2}^{R}\right)},\\ I_{1}= & z_{1}D_{1}J_{11}+z_{2}D_{2}J_{21}\\ = & z_{1}D_{1}\frac{A(z_{2}\left(1-B\right)\lambda +1)}{\left(z_{1}-z_{2}\right)H\left(1\right)}\left(z_{1}\lambda +1\right)+z_{2}D_{2}\frac{A(z_{1}\left(1-B\right)\lambda +1)}{\left(z_{2}-z_{1}\right)H\left(1\right)}\left(z_{2}\lambda +1\right). \end{array} \end{array}$$

Note that the assumption (5) implies that, for fixed $L_{1}$ and $R_{1}$,

$$\begin{array}{cc} c_{1}^{L}= & \sigma^{\frac{z_{1}}{z_{1}-z_{2}}}L_{1},\;c_{1}^{R}=\rho^{\frac{z_{1}}{z_{1}-z_{2}}}R_{1},\;c_{2}^{L}=-\frac{z_{1}}{z_{2}}\sigma^{\frac{z_{1}}{z_{1}-z_{2}}}L_{1},\;c_{2}^{R}=-\frac{z_{1}}{z_{2}}\rho^{\frac{z_{1}}{z_{1}-z_{2}}}R_{1},\\ \lambda (\sigma ,\rho )= & \frac{V-\frac{1}{z_{1}-z_{2}}\left(\ln\sigma -\ln\rho \right)}{\theta (\sigma ,\rho )},\;A\left(\sigma ,\rho \right)=\frac{\left(\alpha -\beta \right){\left(\sigma^{\frac{z_{1}}{z_{1}-z_{2}}}L_{1}-\rho^{\frac{z_{1}}{z_{1}-z_{2}}}R_{1}\right)}^{2}}{\theta (\sigma ,\rho )\omega (\beta ;\sigma ,\rho )\omega (\alpha ;\sigma ,\rho )},\\ B(\sigma ,\rho )= & \theta (\sigma ,\rho )\omega (\beta ;\sigma ,\rho )\omega (\alpha ;\sigma ,\rho )\frac{\ln\omega (\beta ;\sigma ,\rho )-\ln\omega (\alpha ;\sigma ,\rho )}{\left(\alpha -\beta \right){\left(\sigma^{\frac{z_{1}}{z_{1}-z_{2}}}L_{1}-\rho^{\frac{z_{1}}{z_{1}-z_{2}}}R_{1}\right)}^{2}}, \end{array}$$

where

(16) $$\begin{array}{c} \begin{array}{cc} \omega (x;\sigma ,\rho )= & \left(1-x\right)\sigma^{\frac{z_{1}}{z_{1}-z_{2}}}L_{1}+x\rho^{\frac{z_{1}}{z_{1}-z_{2}}}R_{1},\\ \theta (\sigma ,\rho )= & \frac{z_{1}}{z_{1}-z_{2}}\left(\ln\sigma -\ln\rho \right)+\ln L_{1}-\ln R_{1}. \end{array} \end{array}$$

For convenience, we define five functions $P_{00}=P_{00}\left(\sigma ,\rho \right),\;P_{01}=P_{01}\left(\sigma ,\rho \right),$ $P_{10}=P_{10}\left(\sigma ,\rho \right),$ $P_{11}=P_{11}\left(\sigma ,\rho \right)$ and $P_{12}=P_{12}\left(\sigma ,\rho \right)$ by

(17) $$\begin{array}{c} \begin{array}{cc} P_{00} & =\frac{z_{1}\left(\sigma^{\frac{z_{1}}{z_{1}-z_{2}}}L_{1}-\rho^{\frac{z_{1}}{z_{1}-z_{2}}}R_{1}\right)}{H(1)\theta (\sigma ,\rho )}\left(\left(D_{1}-D_{2}\right)\left(\ln L_{1}-\ln R_{1}\right)-D_{2}\left(\ln\sigma -\ln\rho \right)\right),\\ P_{01} & =\frac{z_{1}\left(z_{1}D_{1}-z_{2}D_{2}\right)\left(\sigma^{\frac{z_{1}}{z_{1}-z_{2}}}L_{1}-\rho^{\frac{z_{1}}{z_{1}-z_{2}}}R_{1}\right)}{H(1)\theta (\sigma ,\rho )},\\ P_{10} & =\frac{A}{\left(z_{1}-z_{2}\right)H\left(1\right)}[\frac{z_{1}z_{2}\left(z_{1}D_{1}-z_{2}D_{2}\right)\left(1-B\right){\left(\ln\sigma -\ln\rho \right)}^{2}}{{\left(z_{1}-z_{2}\right)}^{2}\theta^{2}\left(\sigma ,\rho \right)}\\ & -\frac{\left(z_{1}z_{2}\left(D_{1}-D_{2}\right)\left(1-B\right)+z_{1}^{2}D_{1}-z_{2}^{2}D_{2}\right)\left(\ln\sigma -\ln\rho \right)}{\left(z_{1}-z_{2}\right)\theta \left(\sigma ,\rho \right)}+z_{1}D_{1}-z_{2}D_{2}],\\ P_{11} & =\frac{A}{\left(z_{1}-z_{2}\right)H\left(1\right)}[-\frac{2z_{1}z_{2}\left(z_{1}D_{1}-z_{2}D_{2}\right)\left(1-B\right)\left(\ln\sigma -\ln\rho \right)}{\left(z_{1}-z_{2}\right)\theta^{2}\left(\sigma ,\rho \right)}\\ & +\frac{z_{1}z_{2}\left(D_{1}-D_{2}\right)\left(1-B\right)+z_{1}^{2}D_{1}-z_{2}^{2}D_{2}}{\theta (\sigma ,\rho )}],\\ P_{12} & =\frac{z_{1}z_{2}\left(z_{1}D_{1}-z_{2}D_{2}\right)\left(1-B\right)A}{\left(z_{1}-z_{2}\right)H\left(1\right)\theta^{2}\left(\sigma ,\rho \right)}. \end{array} \end{array}$$

Then, $I_{0}$ and $I_{1}$ can be rewritten as

(18) $$\begin{array}{c} \begin{array}{cc} I_{0}\left(V;\sigma ,\rho \right)= & P_{00}\left(\sigma ,\rho \right)+P_{01}\left(\sigma ,\rho \right)V,\\ I_{1}\left(V;\sigma ,\rho \right)= & P_{10}\left(\sigma ,\rho \right)+P_{11}\left(\sigma ,\rho \right)V+P_{12}\left(\sigma ,\rho \right)V^{2}. \end{array} \end{array}$$

Recall that our main interest in this work is to examine the qualitative properties of the I–V relations close to the state of electroneutrality, more precisely, based on our set-ups, it is the case as (σ,ρ)→(1,1). We now employ the regular perturbation analysis and expand $I_{k}\left(V;\sigma ,\rho \right)$ for k=0,1 at $\left(\sigma^{*},\rho^{*}\right)=\left(1,1\right)$ up to the first order and neglect higher orders. Careful calculations give

(19) $$\begin{array}{c} \begin{array}{cc} I_{0}\left(V;\sigma ,\rho \right)= & P_{00}\left(1,1\right)+\frac{\partial P_{00}}{\partial \sigma }\left(1,1\right)\left(\sigma -1\right)+\frac{\partial P_{00}}{\partial \rho }\left(1,1\right)\left(\rho -1\right)+[P_{01}\left(1,1\right)\\ & +\frac{\partial P_{01}}{\partial \sigma }\left(1,1\right)\left(\sigma -1\right)+\frac{\partial P_{01}}{\partial \rho }\left(1,1\right)\left(\rho -1\right)]V,\\ I_{1}\left(V;\sigma ,\rho \right)= & P_{10}\left(1,1\right)+\frac{\partial P_{10}}{\partial \sigma }\left(1,1\right)\left(\sigma -1\right)+\frac{\partial P_{10}}{\partial \rho }\left(1,1\right)\left(\rho -1\right)+[P_{11}\left(1,1\right)\\ & +\frac{\partial P_{11}}{\partial \sigma }\left(1,1\right)\left(\sigma -1\right)+\frac{\partial P_{11}}{\partial \rho }\left(1,1\right)\left(\rho -1\right)]V+[P_{12}\left(1,1\right)\\ & +\frac{\partial P_{12}}{\partial \sigma }\left(1,1\right)\left(\sigma -1\right)+\frac{\partial P_{12}}{\partial \rho }\left(1,1\right)\left(\rho -1\right)]V^{2}, \end{array} \end{array}$$

where

$$\begin{array}{cc} \; & P_{00}\left(1,1\right)=\frac{z_{1}\left(D_{1}-D_{2}\right)}{H(1)}\left(L_{1}-R_{1}\right),\\ \; & \frac{\partial P_{00}}{\partial \sigma }\left(1,1\right)=-\frac{z_{1}\left(z_{1}D_{1}-z_{2}D_{2}\right)}{\left(z_{1}-z_{2}\right)H\left(1\right)}\left(f_{0}\left(L_{1},R_{1}\right)-\frac{z_{1}\left(D_{1}-D_{2}\right)}{z_{1}D_{1}-z_{2}D_{2}}L_{1}\right),\\ \; & \frac{\partial P_{00}}{\partial \rho }\left(1,1\right)=\frac{z_{1}\left(z_{1}D_{1}-z_{2}D_{2}\right)}{\left(z_{1}-z_{2}\right)H\left(1\right)}\left(f_{0}\left(L_{1},R_{1}\right)-\frac{z_{1}\left(D_{1}-D_{2}\right)}{z_{1}D_{1}-z_{2}D_{2}}R_{1}\right), \end{array}$$

$$\begin{array}{cc} \; & P_{01}\left(1,1\right)=\frac{z_{1}\left(z_{1}D_{1}-z_{2}D_{2}\right)}{H(1)}f_{0}\left(L_{1},R_{1}\right),\\ \; & \frac{\partial P_{01}}{\partial \sigma }\left(1,1\right)=-\frac{z_{1}^{2}\left(z_{1}D_{1}-z_{2}D_{2}\right)\left(f_{0}\left(L_{1},R_{1}\right)-L_{1}\right)}{\left(z_{1}-z_{2}\right)H\left(1\right)\left(\ln L_{1}-\ln R_{1}\right)},\\ \; & \frac{\partial P_{01}}{\partial \rho }\left(1,1\right)=\frac{z_{1}^{2}\left(z_{1}D_{1}-z_{2}D_{2}\right)\left(f_{0}\left(L_{1},R_{1}\right)-R_{1}\right)}{\left(z_{1}-z_{2}\right)H\left(1\right)\left(\ln L_{1}-\ln R_{1}\right)},\\ \; & P_{10}\left(1,1\right)=\frac{\left(z_{1}D_{1}-z_{2}D_{2}\right)A\left(1,1\right)}{\left(z_{1}-z_{2}\right)H\left(1\right)},\\ \; & \frac{\partial P_{10}}{\partial \sigma }\left(1,1\right)=\frac{1}{\left(z_{1}-z_{2}\right)H\left(1\right)}(-\frac{z_{1}z_{2}\left(D_{1}-D_{2}\right)\left(1-B\left(1,1\right)\right)+z_{1}^{2}D_{1}-z_{2}^{2}D_{2}}{\left(z_{1}-z_{2}\right)\left(\ln L_{1}-\ln R_{1}\right)}A\left(1,1\right)\\ & \;+\left(z_{1}D_{1}-z_{2}D_{2}\right)\frac{\partial A}{\partial \sigma }\left(1,1\right)),\\ \; & \frac{\partial P_{10}}{\partial \rho }\left(1,1\right)=\frac{1}{\left(z_{1}-z_{2}\right)H\left(1\right)}(\frac{z_{1}z_{2}\left(D_{1}-D_{2}\right)\left(1-B\left(1,1\right)\right)+z_{1}^{2}D_{1}-z_{2}^{2}D_{2}}{\left(z_{1}-z_{2}\right)\left(\ln L_{1}-\ln R_{1}\right)}A\left(1,1\right)\\ & \;+\left(z_{1}D_{1}-z_{2}D_{2}\right)\frac{\partial A}{\partial \rho }\left(1,1\right)),\\ \; & P_{11}\left(1,1\right)=\frac{z_{1}z_{2}\left(D_{1}-D_{2}\right)\left(1-B\left(1,1\right)\right)+z_{1}^{2}D_{1}-z_{2}^{2}D_{2}}{\left(z_{1}-z_{2}\right)H\left(1\right)\left(\ln L_{1}-\ln R_{1}\right)}A\left(1,1\right),\\ \; & \frac{\partial P_{11}}{\partial \sigma }\left(1,1\right)=\frac{1}{\left(z_{1}-z_{2}\right)H\left(1\right)}[\frac{z_{1}z_{2}\left(D_{1}-D_{2}\right)\left(1-B\left(1,1\right)\right)+z_{1}^{2}D_{1}-z_{2}^{2}D_{2}}{\ln L_{1}-\ln R_{1}}\frac{\partial A}{\partial \sigma }\left(1,1\right)\\ & \;-z_{1}(\frac{z_{2}\left(1-B\left(1,1\right)\right)\left(z_{1}\left(D_{1}-D_{2}\right)+2\left(z_{1}D_{1}-z_{2}D_{2}\right)\right)+z_{1}^{2}D_{1}-z_{2}^{2}D_{2}}{\left(z_{1}-z_{2}\right){\left(\ln L_{1}-\ln R_{1}\right)}^{2}}\\ & \;+\frac{z_{2}\left(D_{1}-D_{2}\right)}{\ln L_{1}-\ln R_{1}}\frac{\partial B}{\partial \sigma }\left(1,1\right))A\left(1,1\right)],\\ \; & \frac{\partial P_{11}}{\partial \rho }\left(1,1\right)=\frac{1}{\left(z_{1}-z_{2}\right)H\left(1\right)}(\frac{z_{1}z_{2}\left(D_{1}-D_{2}\right)\left(1-B\left(1,1\right)\right)+z_{1}^{2}D_{1}-z_{2}^{2}D_{2}}{\ln L_{1}-\ln R_{1}}\frac{\partial A}{\partial \rho }\left(1,1\right)\\ & \;+z_{1}[\frac{z_{2}\left(1-B\left(1,1\right)\right)\left(z_{1}\left(D_{1}-D_{2}\right)+2\left(z_{1}D_{1}-z_{2}D_{2}\right)\right)+z_{1}^{2}D_{1}-z_{2}^{2}D_{2}}{\left(z_{1}-z_{2}\right){\left(\ln L_{1}-\ln R_{1}\right)}^{2}}\\ & \;-\frac{z_{2}\left(D_{1}-D_{2}\right)}{\ln L_{1}-\ln R_{1}}\frac{\partial B}{\partial \rho }\left(1,1\right))A\left(1,1\right)],\\ \; & P_{12}\left(1,1\right)=\frac{z_{1}z_{2}\left(z_{1}D_{1}-z_{2}D_{2}\right)\left(1-B\left(1,1\right)\right)A\left(1,1\right)}{\left(z_{1}-z_{2}\right)H\left(1\right){\left(\ln L_{1}-\ln R_{1}\right)}^{2}},\\ \; & \frac{\partial P_{12}}{\partial \sigma }\left(1,1\right)=\frac{z_{1}z_{2}\left(z_{1}D_{1}-z_{2}D_{2}\right)}{\left(z_{1}-z_{2}\right)H\left(1\right)}[\frac{\left(1-B\left(1,1\right)\right)\frac{\partial A}{\partial \sigma }\left(1,1\right)-\frac{\partial B}{\partial \sigma }\left(1,1\right)A\left(1,1\right)}{{\left(\ln L_{1}-\ln R_{1}\right)}^{2}}\\ & \;-\frac{2z_{1}\left(1-B\left(1,1\right)\right)A\left(1,1\right)}{\left(z_{1}-z_{2}\right){\left(\ln L_{1}-\ln R_{1}\right)}^{3}}],\\ \; & \frac{\partial P_{12}}{\partial \rho }\left(1,1\right)=\frac{z_{1}z_{2}\left(z_{1}D_{1}-z_{2}D_{2}\right)}{\left(z_{1}-z_{2}\right)H\left(1\right)}[\frac{\left(1-B\left(1,1\right)\right)\frac{\partial A}{\partial \rho }\left(1,1\right)-\frac{\partial B}{\partial \rho }\left(1,1\right)A\left(1,1\right)}{{\left(\ln L_{1}-\ln R_{1}\right)}^{2}}\\ & \;+\frac{2z_{1}\left(1-B\left(1,1\right)\right)A\left(1,1\right)}{\left(z_{1}-z_{2}\right){\left(\ln L_{1}-\ln R_{1}\right)}^{3}}]. \end{array}$$

Here

$$\begin{array}{cc} f_{0}\left(L_{1},R_{1}\right)= & \frac{L_{1}-R_{1}}{\ln L_{1}-\ln R_{1}},\;A\left(1,1\right)=\frac{\left(\alpha -\beta \right)\left(L_{1}-R_{1}\right)}{\omega (\beta ;1,1)\omega (\alpha ;1,1)}f_{0}\left(L_{1},R_{1}\right),\\ B(1,1)= & \omega (\beta ;1,1)\omega (\alpha ;1,1)\frac{\ln\omega (\beta ;1,1)-\ln\omega (\alpha ;1,1)}{\left(\alpha -\beta \right)\left(L_{1}-R_{1}\right)f_{0}\left(L_{1},R_{1}\right)},\\ \frac{\partial A}{\partial \sigma }\left(1,1\right)= & \frac{z_{1}\left(\alpha -\beta \right)f_{0}\left(L_{1},R_{1}\right)}{\left(z_{1}-z_{2}\right)\omega \left(\alpha ;1,1\right)\omega \left(\beta ;1,1\right)}[2L_{1}-f_{0}\left(L_{1},R_{1}\right)\\ & \;-\frac{\left(1-\beta )\omega (\alpha ;1,1)+(1-\alpha )\omega (\beta ;1,1\right)}{\omega (\alpha ;1,1)\omega (\beta ;1,1)}L_{1}\left(L_{1}-R_{1}\right)],\\ \frac{\partial A}{\partial \rho }\left(1,1\right)= & \frac{z_{1}\left(\alpha -\beta \right)f_{0}\left(L_{1},R_{1}\right)}{\left(z_{1}-z_{2}\right)\omega \left(\alpha ;1,1\right)\omega \left(\beta ;1,1\right)}[-2R_{1}+f_{0}\left(L_{1},R_{1}\right)\\ & -\frac{\beta \omega (\alpha ;1,1)+\alpha \omega (\beta ;1,1)}{\omega (\alpha ;1,1)\omega (\beta ;1,1)}R_{1}\left(L_{1}-R_{1}\right)],\\ \frac{\partial B}{\partial \sigma }\left(1,1\right)= & \frac{1}{A^{2}\left(1,1\right)}[\frac{z_{1}\left(\alpha -\beta \right)L_{1}R_{1}A\left(1,1\right)}{\left(z_{1}-z_{2}\right)\omega \left(\alpha ;1,1\right)\omega \left(\beta ;1,1\right)}\\ & -\left(\ln\omega (\beta ;1,1)-\ln\omega (\alpha ;1,1)\right)\frac{\partial A(\sigma ,\rho )}{\partial \sigma }\left(1,1\right)],\\ \frac{\partial B}{\partial \rho }\left(1,1\right)= & -\frac{1}{A^{2}\left(1,1\right)}[\frac{z_{1}\left(\alpha -\beta \right)L_{1}R_{1}A\left(1,1\right)}{\left(z_{1}-z_{2}\right)\omega \left(\alpha ;1,1\right)\omega \left(\beta ;1,1\right)}\\ & +\left(\ln\omega (\beta ;1,1)-\ln\omega (\alpha ;1,1)\right)\frac{\partial A(\sigma ,\rho )}{\partial \rho }\left(1,1\right)], \end{array}$$

where $\omega \left(\alpha ;1,1\right)=\left(1-\alpha \right)L_{1}+\alpha R_{1}$ and $\omega \left(\beta ;1,1\right)=\left(1-\beta \right)L_{1}+\beta R_{1}.$

## 3. Results

In this section, based on the regular perturbation analysis provided in Section 2.2, we further examine the qualitative properties of ionic flows with boundary layers. To provide more intuitive illustrations of the analytical results obtained in Section 3.1 and Section 3.2, numerical simulations under different set-ups are performed in Section 3.3.

To examine the effects on the I–V relations from the boundary layers, we consider

(20) $$\begin{array}{c} I_{0d}:=I_{0}\left(V;\sigma ,\rho \right)-I_{0}\left(V;1,1\right)\;\mathrm{and}\;I_{1d}:=I_{1}\left(V;\sigma ,\rho \right)-I_{1}\left(V;1,1\right), \end{array}$$

respectively. Correspondingly, one has

(21) $$\begin{array}{c} I_{d}=I_{0d}+QI_{1d}+o\left(Q\right). \end{array}$$

From (19), one has

(22) $$\begin{array}{c} \begin{array}{cc} I_{0d}= & \frac{\partial P_{00}}{\partial \sigma }\left(1,1\right)\left(\sigma -1\right)+\frac{\partial P_{00}}{\partial \rho }\left(1,1\right)\left(\rho -1\right)+[\frac{\partial P_{01}}{\partial \sigma }\left(1,1\right)\left(\sigma -1\right)\\ & +\frac{\partial P_{01}}{\partial \rho }\left(1,1\right)\left(\rho -1\right)]V,\\ I_{1d}= & \frac{\partial P_{10}}{\partial \sigma }\left(1,1\right)\left(\sigma -1\right)+\frac{\partial P_{10}}{\partial \rho }\left(1,1\right)\left(\rho -1\right)+[\frac{\partial P_{11}}{\partial \sigma }\left(1,1\right)\left(\sigma -1\right)\\ & +\frac{\partial P_{11}}{\partial \rho }\left(1,1\right)\left(\rho -1\right)]V+\left[\frac{\partial P_{12}}{\partial \sigma }\left(1,1\right)\left(\sigma -1\right)+\frac{\partial P_{12}}{\partial \rho }\left(1,1\right)\left(\rho -1\right)\right]V^{2}. \end{array} \end{array}$$

### 3.1. Analysis on $I_{0d}$

Note that $I_{0d}$ is a linear function in the potential *V*. The sign of the slope is of particular interest for us, and the following result can be established.

For $t=L_{1}/R_{1}$, direct computation gives

$$\begin{array}{c} \frac{\partial P_{01}}{\partial \sigma }\left(1,1\right)\left(\sigma -1\right)+\frac{\partial P_{01}}{\partial \rho }\left(1,1\right)\left(\rho -1\right)=\frac{z_{1}^{2}\left(z_{1}D_{1}-z_{2}D_{2}\right)R_{1}}{\left(z_{1}-z_{2}\right)H\left(1\right)\ln^{2}t}h\left(t\right), \end{array}$$

where h(t)=(σ−1)tlnt+(1−ρ)lnt+(ρ−σ)(t−1). For the function h(t), one has

**Lemma** **2.** *Assume (σ,ρ)→(1,1) with σ>ρ and $t=L_{1}/R_{1}>1$. One has*

(i) *h(t)>0 if σ+ρ>2 with σ>1;*
(ii) *h(t)<0 if σ+ρ<2 with σ<1;*
(iii) There exists a unique $t^{*}>1$ such that for $\left(\sigma ,\rho \right)\to \left(1^{+},1^{-}\right)$ with σ+ρ<2, one has h(t)<0 if $1<t<t^{*}$ and h(t)>0 if $t>t^{*}$. In particular, $t^{*}$ is the root of h(t)=0.

**Proof.**  The proof is straightforward, and we omit it here. □

It follows from Lemma 2 that

**Theorem** **1.** *Assume (σ,ρ)→(1,1) with σ>ρ and $t=L_{1}/R_{1}>1$. One has*

(i) *$I_{0d}$ is increasing in the potential V if one of the following conditions holds*
  (i1) *σ+ρ>2 with σ>1;*
  (i2) $t>t^{*}$ with σ+ρ<2 and σ>1.
*Furthermore, there exists a unique zero $V_{01}^{d}$ of $I_{0d}\left(V;\sigma ,\rho \right)=0$ such that*
*$I_{0d}\left(V;\sigma ,\rho \right)>0$ (respectively, $I_{0d}\left(V;\sigma ,\rho \right)<0$) if $V>V_{01}^{d}$ (respectively, $V<V_{01}^{d}$), equivalently, the effect from boundary layers enhances (respectively, reduces) $I_{0}$ for $V>V_{01}^{d}$ (respectively, $V<V_{01}^{d}$).*
(ii) *$I_{0d}$ is decreasing in the potential V if one of the following conditions holds*
  (ii1) *σ+ρ<2 with σ<1;*
  (ii2) $t<t^{*}$ with σ+ρ<2 and σ>1.
*Furthermore, there exists a unique zero $V_{02}^{d}$ of $I_{0d}\left(V;\sigma ,\rho \right)=0$ such that*
*$I_{0d}\left(V;\sigma ,\rho \right)>0$ (respectively, $I_{0d}\left(V;\sigma ,\rho \right)<0$) if $V<V_{02}^{d}$ (respectively, $V>V_{02}^{d}$), equivalently, the effect from boundary layers enhances (respectively, reduces) $I_{0}$ for $V<V_{02}^{d}$ (respectively, $V>V_{02}^{d}$).*

### 3.2. Analysis of $I_{1d}$

We first consider the sign of the coefficient of $V^{2}$ in $I_{1d}\left(V\right)$. For convenience, we define $g\left(\beta \right)=\frac{\partial P_{12}}{\partial \sigma }\left(1,1\right)\left(\sigma -1\right)+\frac{\partial P_{12}}{\partial \rho }\left(1,1\right)\left(\rho -1\right)$. For $t=L_{1}/R_{1}$, one has

$$\begin{array}{cc} g(\beta ) & =\frac{{z_{1}}^{2}z_{2}\left(z_{1}D_{1}-z_{2}D_{2}\right)}{{\left(z_{1}-z_{2}\right)}^{2}\omega_{1}\left(\alpha ;1,1\right)\omega_{1}\left(\beta ;1,1\right)H\left(1\right)\ln^{3}t}g_{1}\left(\beta \right), \end{array}$$

where

$$\begin{array}{cc} g_{1}\left(\beta \right) & =2\omega_{1}\left(\alpha ;1,1\right)\omega_{1}\left(\beta ;1,1\right)\left(\sigma -\rho \right)\ln\frac{\omega_{1}\left(\beta ;1,1\right)}{\omega_{1}\left(\alpha ;1,1\right)}\\ \; & +\left(\alpha -\beta \right)[t\left(\rho -\sigma \right)\ln t+\left(t-1\right)(2t\left(\sigma -1\right)-2\left(\rho -1\right)+\frac{3(t-1)(\rho -\sigma )}{\ln t}\\ \; & -\frac{t-1}{\omega_{1}\left(\alpha ;1,1\right)\omega_{1}\left(\beta ;1,1\right)}(t\left(\sigma -1\right)\left[\left(1-\beta \right)\omega_{1}\left(\alpha ;1,1\right)+\left(1-\alpha \right)\omega_{1}\left(\beta ;1,1\right)\right]\\ \; & +\left(\rho -1\right)[\beta \omega_{1}\left(\alpha ;1,1\right)+\alpha \omega_{1}\left(\beta ;1,1\right)]))],\\ \omega_{1}(\alpha ; & 1,1)=\left(1-\alpha \right)t+\alpha ,\;\omega_{1}\left(\beta ;1,1\right)=\left(1-\beta \right)t+\beta . \end{array}$$

**Lemma** **3.** *Assume (σ,ρ)→(1,1) with σ>ρ and $t=L_{1}/R_{1}>1$. Let $\alpha_{1}=\frac{t-\sqrt{t}}{t-1}$, one has*

(i) *If $\alpha \in (0,\alpha_{3})$, then g(β)>0;*
(ii) *If $\alpha \in [\alpha_{3},\alpha_{1})$, then g(β)<0;*
(iii) *If $\alpha \in [\alpha_{1},\alpha_{4})$, then there exists a unique $\beta_{1}>\alpha$ such that g(β)<0 for $\beta \in (\alpha ,\beta_{1})$ and g(β)>0 for $\beta \in (\beta_{1},1)$;*
(iv) *If $\alpha \in [\alpha_{4},1)$, then g(β)>0.*

*Here, $\alpha_{3}$ and $\alpha_{4}$ are two roots of $g_{2}\left(\alpha \right)=0$ with*

$$\begin{array}{cc} g_{2}\left(\alpha \right) & =\left(1-t\right)(2t\ln t+3\left(1-t\right)\omega_{1}\left(\alpha ;1,1\right))+t\omega_{1}\left(\alpha ;1,1\right)\ln^{2}t-2\left(t-1\right)\omega_{1}^{2}\left(\alpha ;1,1\right)\ln t. \end{array}$$

*They are given by*

$$\begin{array}{cc} \alpha_{3} & =\frac{-3{\left(1-t\right)}^{2}-t\ln^{2}t+4t\left(t-1\right)\ln t-\sqrt{p(t)}}{4{\left(t-1\right)}^{2}\ln t},\\ \alpha_{4} & =\frac{-3{\left(1-t\right)}^{2}-t\ln^{2}t+4t\left(t-1\right)\ln t+\sqrt{p(t)}}{4{\left(t-1\right)}^{2}\ln t}, \end{array}$$

*where $p\left(t\right)=9{\left(t-1\right)}^{4}+t^{2}\ln^{4}t-10t{\left(t-1\right)}^{2}\ln^{2}t.$*

**Proof.**  It is clear that g(β) has the opposite sign as that of $g_{1}\left(\beta \right)$. Note that

$$\begin{array}{cc} \lim_{\beta \to \alpha }g_{1}\left(\beta \right) & =0,\;\lim_{\beta \to \alpha }g_{1}^{\prime }\left(\beta \right)=\frac{\sigma -\rho }{\omega_{1}\left(\alpha ;1,1\right)\ln t}g_{2}\left(\alpha \right),\;\lim_{\beta \to \alpha }g_{1}^{\prime \prime }\left(\beta \right)=\frac{2\left(\sigma -\rho \right){\left(t-1\right)}^{2}}{\omega_{1}^{2}\left(\alpha ;1,1\right)}g_{3}\left(\alpha \right), \end{array}$$

where $g_{3}\left(\alpha \right)=\omega_{1}^{2}\left(\alpha ;1,1\right)-t.$

For t>1, $g_{3}\left(\alpha \right)$ is a quadratic function in α, and concave upward, it follows that $g_{3}\left(\alpha \right)=0$ has two roots given by $\alpha_{1}$ and $\alpha_{2}=\frac{t+\sqrt{t}}{t-1}>1$. For (σ,ρ)→(1,1), σ>ρ and $t=L_{1}/R_{1}>1$, if $\alpha \in (0,\alpha_{1}]$, then $g_{3}\left(\alpha \right)\geq 0$ and $\lim_{\beta \to \alpha }g_{1}^{\prime \prime }\left(\beta \right)\geq 0$; if $\alpha \in (\alpha_{1},1)$, then $g_{3}\left(\alpha \right)<0$ and $\lim_{\beta \to \alpha }g_{1}^{\prime \prime }\left(\beta \right)<0$.

For t>1, $g_{2}\left(\alpha \right)$ is a quadratic function in α, and concave downward, it follows that $g_{2}\left(\alpha \right)=0$ has two roots given by $\alpha_{3}$ and $\alpha_{4}$. For (σ,ρ)→(1,1), σ>ρ and $t=L_{1}/R_{1}>1$, if $\alpha \in \left(0,\alpha_{3}\right]\cup \left[\alpha_{4},1\right)$, then $g_{2}\left(\alpha \right)\leq 0$ and $\lim_{\beta \to \alpha }g_{1}^{\prime }\left(\beta \right)\leq 0$; if $\alpha \in (\alpha_{3},\alpha_{4})$, then $g_{2}\left(\alpha \right)>0$ and $\lim_{\beta \to \alpha }g_{1}^{\prime }\left(\beta \right)>0$.

Direct computation gives $0<\alpha_{3}<\alpha_{1}<\alpha_{4}<1$, for (σ,ρ)→(1,1), σ>ρ and $t=L_{1}/R_{1}>1$. Since $\lim_{\beta \to \alpha }g_{1}\left(\beta \right)=0$, one has

(i) If $\alpha \in (0,\alpha_{3})$, then $\lim_{\beta \to \alpha }g_{1}^{\prime \prime }\left(\beta \right)>0$ and $\lim_{\beta \to \alpha }g_{1}^{\prime }\left(\beta \right)<0$. We can easily get $g_{1}\left(1\right)<0$, and hence $g_{1}\left(\beta \right)<0$;
(ii) If $\alpha \in [\alpha_{3},\alpha_{1})$, then $\lim_{\beta \to \alpha }g_{1}^{\prime \prime }\left(\beta \right)>0$ and $\lim_{\beta \to \alpha }g_{1}^{\prime }\left(\beta \right)\geq 0$, hence $g_{1}\left(\beta \right)>0$;
(iii) If $\alpha \in [\alpha_{1},\alpha_{4})$, then $\lim_{\beta \to \alpha }g_{1}^{\prime \prime }\left(\beta \right)\leq 0$ and $\lim_{\beta \to \alpha }g_{1}^{\prime }\left(\beta \right)>0$. We can easily get $g_{1}\left(1\right)<0$, and hence, there exists a unique $\beta_{1}>\alpha$ such that $g_{1}\left(\beta \right)>0$ for $\beta \in (\alpha ,\beta_{1})$ and $g_{1}\left(\beta \right)<0$ for $\beta \in (\beta_{1},1)$;
(iv) If $\alpha \in [\alpha_{4},1)$, then $\lim_{\beta \to \alpha }g_{1}^{\prime \prime }\left(\beta \right)<0$ and $\lim_{\beta \to \alpha }g_{1}^{\prime }\left(\beta \right)\leq 0$, hence $g_{1}\left(\beta \right)<0$.

This completes the proof. □

Form Lemma 3, the following result can be established.

**Theorem** **2.** *Assume (σ,ρ)→(1,1) with σ>ρ and $t=L_{1}/R_{1}>1$. There exists a unique critical potential $V_{1}^{c}$ such that*

(i) *For $\alpha \in (0,\alpha_{3})$, one has $I_{1d}\left(V\right)$ increases (respectively, decreases) in the potential V if $V>V_{1}^{c}$ (respectively, $V<V_{1}^{c}$);*
(ii) *For $\alpha \in [\alpha_{3},\alpha_{1})$, one has $I_{1d}\left(V\right)$ increases (respectively, decreases) in the potential V if $V<V_{1}^{c}$ (respectively, $V>V_{1}^{c}$);*
(iii) *For $\alpha \in [\alpha_{1},\alpha_{4})$, then there exists a unique $\beta_{1}>\alpha$ such that $I_{1d}\left(V\right)$ decreases in the potential V for $\beta \in (\alpha ,\beta_{1})$ and increases in V for $\beta \in (\beta_{1},1)$;*
(iv) *For $\alpha \in [\alpha_{4},1)$, $I_{1d}\left(V\right)$ increases in the potential V.*

We now turn to the sign of $I_{1d}\left(V\right)$. We first consider the sign of $g_{4}\left(\alpha \right)$, which will be used in the proof of Theorem 3. Here, $g_{4}\left(\alpha \right)$ is defined by

$$\begin{array}{cc} \; & g_{4}\left(\alpha \right)=\left(4t^{2}\ln^{2}t-8t\left(t-1\right)\omega_{1}\left(\alpha ;1,1\right)\ln t+3{\left(t-1\right)}^{2}\omega_{1}^{2}\left(\alpha ;1,1\right)\right)[{\left(z_{1}^{2}D_{1}-z_{2}^{2}D_{2}\right)}^{2}\\ & +z_{1}z_{2}\left(\alpha -\gamma \left(t\right)\right)(8z_{1}z_{2}D_{1}D_{2}-2\left(z_{1}^{2}D_{1}+z_{2}^{2}D_{2}\right)\left(D_{1}+D_{2}\right))\ln t\\ & +z_{1}^{2}z_{2}^{2}{\left(D_{1}-D_{2}\right)}^{2}{\left(\alpha -\gamma \left(t\right)\right)}^{2}\ln^{2}t]+{\left(t-1\right)}^{2}\omega_{1}^{2}\left(\alpha ;1,1\right)(z_{1}^{2}D_{1}-z_{2}^{2}D_{2}\\ & +z_{2}\left(2z_{2}D_{2}-z_{1}D_{1}-z_{1}D_{2}\right)\left(\alpha -\gamma \left(t\right)\right)\ln t{)}^{2}+2z_{2}\left(t\ln t-\left(t-1\right)\omega_{1}\left(\alpha ;1,1\right)\right)\ln t\\ & \times \left(\left(z_{1}^{2}D_{1}-z_{2}^{2}D_{2}\right)\left(z_{1}D_{1}+z_{1}D_{2}-2z_{2}D_{2}\right)-z_{1}^{2}z_{2}{\left(D_{1}-D_{2}\right)}^{2}\left(\alpha -\gamma \left(t\right)\right)\ln t\right)\omega_{1}^{2}\left(\alpha ;1,1\right). \end{array}$$

For the function $g_{4}\left(\alpha \right)$, the following result can be established.

**Lemma** **4.** Assume $t=L_{1}/R_{1}\in \left(1,2\right)$ and γ(t) be as in (14). For the function $g_{4}\left(\alpha \right)$, there exists a unique zero $\alpha^{*}$ such that $g_{4}\left(\alpha \right)>0$ for $\alpha <\alpha^{*}$, and $g_{4}\left(\alpha \right)<0$ for $\alpha >\alpha^{*}.$

**Proof.**  The discussion is then straightforward, and we omit it here. □

**Remark** **1.** *The function $g_{4}\left(\alpha \right)$ defined above is very complicated to analyze. The number of zeros depends on the parameter t sensitively. In this work, we just consider the simplest case.*

If g(β)≠0, then $I_{1d}=0$ is a quadratic equation in *V*, whose discriminant is

$$\begin{array}{cc} \; & \Delta =\frac{\Delta_{1}}{{\left(z_{1}-z_{2}\right)}^{2}H^{2}\left(1\right){\left(\ln L_{1}-\ln R_{1}\right)}^{2}}, \end{array}$$

where $\Delta_{1}=\Delta_{10}+\Delta_{11}+\Delta_{12}+\Delta_{13}+\Delta_{14}$ with

$$\begin{array}{cc} \; & \Delta_{10}=[z_{1}z_{2}\left(1-B\left(1,1\right)\right)(8z_{1}z_{2}D_{1}D_{2}-2\left(z_{1}^{2}D_{1}+z_{2}^{2}D_{2}\right)\left(D_{1}+D_{2}\right))\\ \; & \;+{\left(z_{1}^{2}D_{1}-z_{2}^{2}D_{2}\right)}^{2}+z_{1}^{2}z_{2}^{2}{\left(D_{1}-D_{2}\right)}^{2}{\left(1-B\left(1,1\right)\right)}^{2}]\\ \; & \;\times \left(\frac{\partial A}{\partial \sigma }\left(1,1\right)\left(\sigma -1\right)+\frac{\partial A}{\partial \rho }\left(1,1\right)\left(\rho -1\right)\right)\\ \; & \;\times \left(\frac{\partial A}{\partial \sigma }\left(1,1\right)\left(\sigma -1\right)+\frac{\partial A}{\partial \rho }\left(1,1\right)\left(\rho -1\right)+\frac{2z_{1}\left(\rho -\sigma \right)A\left(1,1\right)}{\left(z_{1}-z_{2}\right)\left(\ln L_{1}-\ln R_{1}\right)}\right),\\ \; & \Delta_{11}=\frac{z_{1}^{2}{\left(\sigma -\rho \right)}^{2}A^{2}\left(1,1\right)}{{\left(z_{1}-z_{2}\right)}^{2}{\left(\ln L_{1}-\ln R_{1}\right)}^{2}}(z_{1}^{2}D_{1}-z_{2}^{2}D_{2}\\ \; & \;+z_{2}\left(1-B\left(1,1\right)\right)\left(2z_{2}D_{2}-z_{1}D_{1}-z_{1}D_{2}\right){)}^{2}, \end{array}$$

$$\begin{array}{cc} \; & \Delta_{12}=z_{1}^{2}z_{2}^{2}{\left(D_{1}-D_{2}\right)}^{2}[\frac{z_{1}\left(\alpha -\beta \right)\left(\sigma -\rho \right)L_{1}R_{1}A\left(1,1\right)}{\left(z_{1}-z_{2}\right)\omega \left(\alpha ;1,1\right)\omega \left(\beta ;1,1\right)}\\ \; & \;-\left(\frac{\partial A}{\partial \sigma }\left(1,1\right)\left(\sigma -1\right)+\frac{\partial A}{\partial \rho }\left(1,1\right)\left(\rho -1\right)\right)\ln\frac{\omega (\beta ;1,1)}{\omega (\alpha ;1,1)}{]}^{2},\\ \; & \Delta_{13}=\frac{2z_{1}z_{2}\left(\rho -\sigma \right)}{\left(z_{1}-z_{2}\right)\left(\ln L_{1}-\ln R_{1}\right)}(-z_{1}^{2}z_{2}{\left(D_{1}-D_{2}\right)}^{2}\left(1-B\left(1,1\right)\right)\\ \; & \;+\left(z_{1}^{2}D_{1}-z_{2}^{2}D_{2}\right)\left(z_{1}D_{1}+z_{1}D_{2}-2z_{2}D_{2}\right))[\frac{z_{1}\left(\alpha -\beta \right)\left(\sigma -\rho \right)L_{1}R_{1}A\left(1,1\right)}{\left(z_{1}-z_{2}\right)\omega \left(\alpha ;1,1\right)\omega \left(\beta ;1,1\right)}\\ \; & \;-\left(\frac{\partial A}{\partial \sigma }\left(1,1\right)\left(\sigma -1\right)+\frac{\partial A}{\partial \rho }\left(1,1\right)\left(\rho -1\right)\right)\ln\frac{\omega (\beta ;1,1)}{\omega (\alpha ;1,1)}],\\ \; & \Delta_{14}=\frac{2z_{1}z_{2}}{A(1,1)}(2{\left(z_{1}D_{1}-z_{2}D_{2}\right)}^{2}-z_{1}z_{2}{\left(D_{1}-D_{2}\right)}^{2}\left(1-B\left(1,1\right)\right)\\ \; & \;-\left(D_{1}-D_{2}\right)\left(z_{1}^{2}D_{1}-z_{2}^{2}D_{2}\right))[\frac{z_{1}\left(\alpha -\beta \right)\left(\sigma -\rho \right)L_{1}R_{1}A\left(1,1\right)}{\left(z_{1}-z_{2}\right)\omega \left(\alpha ;1,1\right)\omega \left(\beta ;1,1\right)}\\ \; & \;-\left(\frac{\partial A}{\partial \sigma }\left(1,1\right)\left(\sigma -1\right)+\frac{\partial A}{\partial \rho }\left(1,1\right)\left(\rho -1\right)\right)\ln\frac{\omega (\beta ;1,1)}{\omega (\alpha ;1,1)}]\\ \; & \;\times \left(\frac{\partial A}{\partial \sigma }\left(1,1\right)\left(\sigma -1\right)+\frac{\partial A}{\partial \rho }\left(1,1\right)\left(\rho -1\right)\right). \end{array}$$

**Theorem** **3.** Assume (σ,ρ)→(1,1) with σ>ρ and $t=L_{1}/R_{1}\in \left(1,2\right)$. With $0<\alpha_{3}<\alpha^{*}<\alpha_{1}<\alpha_{4}<1$, one has

(i) *If $\alpha \in (0,\alpha_{3})$, then Δ>0, and there exist two critical potentials $V_{11}^{d}$ and $V_{12}^{d}$ (assuming $V_{11}^{d}<V_{12}^{d}$ for convenience) such that*
  (i1) *If $V=V_{11}^{d}$ or $V=V_{12}^{d}$, then $I_{1d}\left(V\right)=0$;*
  (i2) *If $V\in (V_{11}^{d},V_{12}^{d})$, then $I_{1d}\left(V\right)<0$;*
  (i3) *If $V\notin (V_{11}^{d},V_{12}^{d})$, then $I_{1d}\left(V\right)>0$;*
(ii) If $\alpha \in (\alpha_{3},\alpha^{*})$, then Δ>0, and there exist two critical potentials $V_{13}^{d}$ and $V_{14}^{d}$ (assuming $V_{13}^{d}<V_{14}^{d}$ for convenience) such that
  (i1) *If $V=V_{13}^{d}$ or $V=V_{14}^{d}$, then $I_{1d}\left(V\right)=0$;*
  (i2) *If $V\in (V_{13}^{d},V_{14}^{d})$, then $I_{1d}\left(V\right)>0$;*
  (i3) *If $V\notin (V_{13}^{d},V_{14}^{d})$, then $I_{1d}\left(V\right)<0$;*
(iii) If $\alpha \in (\alpha^{*},\alpha_{1})$, then Δ<0, and hence $I_{1d}\left(V\right)<0$;
(iv) *If $\alpha \in (\alpha_{1},\alpha_{4})$, then Δ<0, and there exists a unique $\beta_{1}>\alpha$ such that $I_{1d}\left(V\right)<0$ for $\beta \in (\alpha ,\beta_{1})$ and $I_{1d}\left(V\right)>0$ for $\beta \in (\beta_{1},1)$;*
(v) *If $\alpha \in (\alpha_{4},1)$, then Δ<0, and hence $I_{1d}\left(V\right)>0$.*

**Proof.**  It is clear that Δ has the same sign as that of $\Delta_{1}$. For 1<t<2, one has

$$\begin{array}{cc} \; & \lim_{\beta \to \alpha }\Delta_{1}=0,\;\lim_{\beta \to \alpha }\Delta_{1}^{\prime }=0,\\ \; & \lim_{\beta \to \alpha }\Delta_{1}^{\prime \prime }=\frac{2z_{1}^{2}{\left(t-1\right)}^{2}{\left(\sigma -\rho \right)}^{2}}{{\left(z_{1}-z_{2}\right)}^{2}\omega_{1}^{6}\left(\alpha ;1,1\right)\ln^{4}t}g_{4}\left(\alpha \right). \end{array}$$

From Lemma 4, we can easily obtain the sign of $\lim_{\beta \to \alpha }\Delta_{1}^{\prime \prime }$, and hence know the sign of $\Delta_{1}$. □

**Remark** **2.** In Theorem 3, the result established further depends on the order of some critical values related to channel geometry, $\alpha^{*},\alpha_{1},\alpha_{3}$ and $\alpha_{4}$. We demonstrate that the order is not unique, which further depends on the nonlinear interaction between other system parameters, particularly, a,b, the jumping points of the permanent charge, and $L_{1},R_{1}$, the boundary concentrations. However, similar result should be obtained. Moreover, the result stated in Theorem 3 indicates that under different conditions on the channel geometry, the small positive permanent charge $Q_{0}$ can either enhance the current $I_{0d}\left(V;\sigma ,\rho \right)$ or reduce it. Taking the statement (i) as an example, for $\alpha \in (0,\alpha^{*})$ with $V_{11}^{d}<V_{12}^{d}$, one has the small permanent charge $Q_{0}$ enhances $I_{0d}\left(V;\sigma ,\rho \right)$ for $V\in \left(-\infty ,V_{11}^{d}\right)\cup \left(V_{12}^{d},\infty \right)$, while reduces it for $V\in (V_{11}^{d},V_{12}^{d})$. Furthermore, the result stated in Theorem 2 provides an efficient way to adjust the effects from the leading term $I_{1d}$ that contains small permanent charge.

### 3.3. Numerical Simulations

In this part, numerical simulations are performed to provide more intuitive illustrations of some analytical results. To be specific, we numerically identify the critical potentials $V_{01}^{d},V_{11}^{d},V_{12}^{d}$ and $V_{1}^{c}$, which characterized the effects caused by the appearance of boundary layers. To further illustrate the boundary layer effects on ionic flows, we also numerically obtain the zeroth-order (respectively, the first-order) I–V relations in small positive $Q_{0}$ without and with boundary layers, respectively. Corresponding critical potentials for each setup are identified, from which one is able to observe the effects on ionic flows from boundary layers clearly.

To get started, we rewrite the system (7) and (8) as a system of first-order ordinary differential equations. Upon introducing $u=\varepsilon \frac{d}{dx}\phi$, one has

(23) $$\begin{array}{c} \begin{array}{cc} \varepsilon \frac{d}{dx}\phi = & u,\;\frac{\varepsilon }{h(x)}\frac{d}{dx}\left(h\left(x\right)u\right)=-z_{1}c_{1}-z_{2}c_{2}-Q\left(x\right),\\ \varepsilon \frac{d}{dx}c_{1}= & -z_{1}c_{1}u-\varepsilon \frac{J_{1}}{h(x)},\;\varepsilon \frac{d}{dx}c_{2}=-z_{2}c_{2}u-\varepsilon \frac{J_{2}}{h(x)},\;\frac{d}{dx}J_{1}=\frac{d}{dx}J_{2}=0, \end{array} \end{array}$$

with boundary conditions

(24) $$\phi \left(0\right)=V,\;c_{k}\left(0\right)=L_{k};\;\phi \left(1\right)=0,\;c_{k}\left(1\right)=R_{k},\;k=1,2.$$

In our simulations to system (23) and (24), we take $z_{1}=-z_{2}=1,\;L_{1}=12,\;R_{1}=8,\;\varepsilon =0.01,\;Q_{0}=0.008,\;a=0.4,\;b=0.48,$ $D_{1}=2.032,\;D_{2}=1.334$,

$$Q\left(x\right)=\left\{\begin{array}{cc} 0, & 0<x<a,\\ Q_{0}, & a<x<b,\\ 0, & b<x<1, \end{array}\right.\;\mathrm{and}\;h\left(x\right)=\left\{\begin{array}{cc} \pi {\left(-x+r_{0}+a\right)}^{2}, & 0\leq x<a,\\ \pi r_{0}^{2}, & a\leq x<b,\\ \pi {\left(x+r_{0}-b\right)}^{2}, & b\leq x<1. \end{array}\right.$$

We comment that the choice of h(x) is based on the fact that the ion channel is cylinder-like, and the variable cross-section area is chosen to reflect the fact that the channel is not uniform and much narrower in the neck (0.4<x<0.48) than other regions ([11]). We further take $r_{0}=0.5$ and the function h(x) is then continuous at the jumping points a=0.4 and b=0.48. Different models for h(x) may be chosen, and similar numerical results should be obtained.

**Figure 1:**
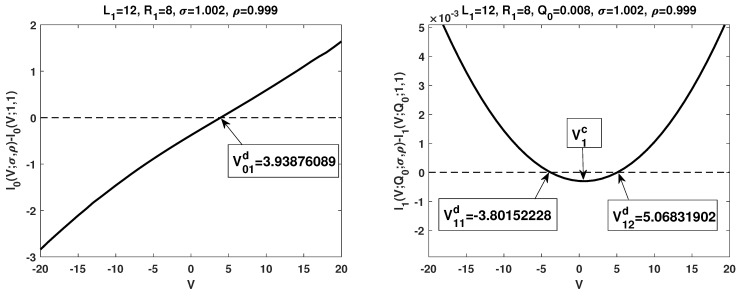
Numerical simulations for effects on I–V relations from boundary layers with small positive permanent charge.

Our numerical simulations show that:

(A) The term $I_{0d}\left(V\right)$ is increasing in the potential *V*, and there exists a unique zero $V_{01}^{d}$ such that $I_{0d}\left(V\right)>0$ for $V>V_{01}^{d}$ while $I_{0d}\left(V\right)<0$ for $V<V_{01}^{d}$. This is consistent with the first statement in Theorem 1 (see the left figure in Figure 1);
(B) The term $I_{1d}\left(V\right)$, as a quadratic function, under our setup, it is concave up with two zeros $V_{11}^{d}$ and $V_{12}^{d}$. At $V_{1}^{c}$, the critical point, $I_{1d}\left(V\right)$ attains its global minimum. Furthermore, $I_{1d}\left(V\right)>0$ (respectively, $I_{1d}\left(V\right)<0$) for $V\in \left(-\infty ,V_{11}^{d}\right)\cup \left(V_{12}^{d},\infty \right)$ (respectively, $V\in (V_{11}^{d},V_{12}^{d})$); $I_{1d}\left(V\right)$ is increasing for $V>V_{1}^{c}$ while it is decreasing for $V<V_{1}^{c}$. The numerical results are consistent with the first statement in Theorem 2 and Theorem 3, respectively (see the right figure in Figure 1).

**Figure 2:**
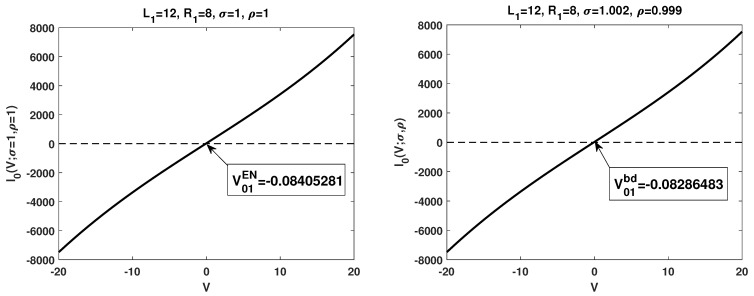
Numerical simulations of $I_{0}\left(V\right)$, the zeroth-order I–V relations in $Q_{0}$ for two different setups. The left figure is under electroneutrality boundary conditions, while the right one is with boundary layers.

**Figure 3:**
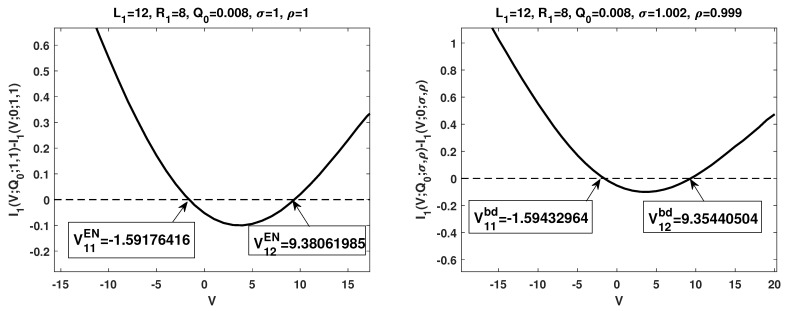
Numerical simulations of $I_{1}\left(V\right)$, the first-order I–V relations in $Q_{0}$ for two different setups. The left figure is under electroneutrality boundary conditions, while the right one is with boundary layers.

To further illustrate the boundary layer effects on the I–V relations, we also performed numerical simulations to the system with electroneutrality boundary concentration conditions (no boundary layers, the left figures in both Figure 2 and Figure 3) and relaxed electroneutrality boundary conditions (appearance of boundary layers, the right figures in both Figure 2 and Figure 3), respectively. In each setup, the corresponding critical potentials are identified. To be specific, $V_{01}^{EN},V_{11}^{EN}$ and $V_{12}^{EN}$ are the critical potentials detected under electroneutrality boundary conditions, while $V_{01}^{bd},\;V_{11}^{bd}$ and $V_{12}^{bd}$ are the ones with boundary layers. From the numerical simulations, one observes $V_{01}^{EN}<V_{01}^{bd},\;V_{11}^{EN}>V_{11}^{bd}$ and $V_{12}^{EN}>V_{12}^{bd}.$ The observation indicates the important role played by the boundary layers. We take $I_{0}\left(V\right)$ as an example. For many studies on PNP type models, electroneutrality boundary conditions are applied, based on our numerical result, this means $I_{0}\left(V;1,1\right)>0$ for $V>V_{01}^{EN}$. However, if the boundary layers are considered, $I_{0}\left(V;\sigma ,\rho \right)<0$ for $V_{01}^{EN}<V<V_{01}^{bd}$, even for (σ,ρ)→(1,1). The dynamics of ionic flows are totally different.

**Remark** **3.** *For our numerical simulation, we only considered the case with $D_{1}>D_{2}$. Interested readers can follow our argument to consider the case with $D_{1}<D_{2}$.*

## 4. Concluding Remarks

We study a one-dimensional steady-state Poisson–Nernst–Planck system with two oppositely charged ion species and small but nonzero permanent charges. The main purpose is to understand the problem from the mathematical point of view, which should provide some insights into related studies of ion channel problems. Particularly, we focus on the qualitative properties of the I–V relations with boundary layers due to the violation of the electroneutrality boundary conditions, which are studied from two directions:

(i) Boundary layer effects on the zeroth-order (in $Q_{0}$) I–V relations in terms of the function $I_{0d}\left(V;\sigma ,\rho \right)$;
(ii) Boundary layer effects on the first-order (in $Q_{0}$) I–V relations in terms of the function $I_{1d}\left(V;\sigma ,\rho \right)$.

Detailed analysis along each direction is provided, which includes the signs of $I_{0d}\left(V;\sigma ,\rho \right)$ and $I_{1d}\left(V;\sigma ,\rho \right)$ and their monotonicity. From the study, one can better understand the mechanism of ionic flows through membrane channels, particularly the internal dynamics of ionic flows, which are non-intuitive and cannot be detected by current technology. Critical potentials that balance the small permanent charge effects on the I–V relations are identified, and their critical roles played in the study of ionic flow properties are characterized. Numerical simulations are performed to provide more intuitive illustrations of the analytical results, and they are consistent. Among others, we find

(I) The monotonicity of $I_{0d}$ depends sensitively on the parameter *t* defined by $L_{1}/R_{1}$ and the boundary layers through the parameters σ and ρ;
(II) The sign of $I_{1d}$ depends sensitively on the interplays among system parameters, particularly, the parameter *t* defined by the ratio $L_{1}/R_{1}$, and the parameter α=H(a)/H(1) representing the channel geometry, while the monotonicity of $I_{1d}$ is only sensitive on α.

Finally, we comment that the setup in this work is relatively simple, and the study in this work is the first step in the analysis of more realistic models. The simple model considered allows us to obtain a more explicit expression of the I–V relations in terms of physical parameters of the problem so that we are able to extract concrete information of the effects from boundary layers and small but nonzero permanent charges. Moreover, the analysis in this simpler setting provides further understanding of the qualitative properties of the I–V relations through membrane channels, and detailed characterization of the nonlinear interplay among different system parameters. The critical potentials identified in this work are critical for one to observe different ionic flow properties through membrane channels. More importantly, some of them can be approximated experimentally.

## Data Availability

Not applicable.
